# The Evidence of SARS-CoV-2 Human-to-Pets Transmission in Household Settings in Bosnia and Herzegovina

**DOI:** 10.3389/fgene.2022.839205

**Published:** 2022-04-26

**Authors:** Sejla Goletic, Teufik Goletic, Adis Softic, Amir Zahirovic, Dunja Rukavina, Aida Kavazovic, Jasmin Omeragic, Sekib Umihanic, Mirsada Hukic

**Affiliations:** ^1^ Veterinary Faculty, University of Sarajevo, Sarajevo, Bosnia and Herzegovina; ^2^ Medical Faculty, University of Tuzla, Tuzla, Bosnia and Herzegovina; ^3^ Clinic for Ear, Nose, Throat Disease, Head Neck Surgery, University Clinical Center Tuzla, Sarajevo, Bosnia and Herzegovina; ^4^ The Academy of Science and Arts of Bosnia and Herzegovina, Sarajevo, Bosnia and Herzegovina

**Keywords:** SARS-CoV-2, one health approach, whole genome sequencing, ELISA, pets

## Abstract

The infection with SARS-CoV-2 virus in cats and dogs raised issue of human-to-animal transmission of SARS-CoV-2 in domestic pets in close contacts with their owners. Our study was designed to research this in the framework of Bosnia and Herzegovina. Using ELISA, AFIAS fluorescent immunoassay, RT-qPCR and WGS on Nanopore MinION platform with ARTIC Network Amplicon sequencing protocol for SARS-CoV-2, we showed that three out of thirteen dogs and one out of five cats from the households with confirmed human cases of COVID-19 in Bosnia-Herzegovina were infected with SARS-CoV-2. The high viral RNA load was detected in samples collected from a 4-year-old male Havanese (Ct = 12.52), a 6-year-old German Shepherd (Ct = 21.36) and a 9-year-old female American Staffordshire terrier (Ct = 25.74). The antibody response in dogs and one cat was observed. The viral genetic sequences from dogs were identical to the sequences detected in the owners suggesting the human-to-animal transmission of the virus. These findings, especially the low initial Ct values detected, from the public health perspective additionally stress the need for precautionary measures to protect both humans and animals.

## Introduction

A novel coronavirus disease 2019 (COVID-19) caused by severe acute respiratory syndrome coronavirus 2 (SARS-CoV-2) was first confirmed in Wuhan, the People’s Republic of China in December 2019 (Zhu et al., 2020), and has since been reported in over 166 million cases worldwide (WHO Coronavirus (COVID-19) Dashboard, available at https://covid19.who.int/; accessed 22 May 2021). The viral genome has proven to be susceptible to frequent mutations, which has, so far, given rise to five Variants of Concern (VOC) and two Variants of Interest (VOI) (World Health Organisation, 2021). SARS-CoV-2 has also shown the ability to infect household pets, which entails the need for handling such cases (Kiros et al., 2020). These facts, as well as the results of previous studies that demonstrated the need for intensive surveillance of SARS-CoV-2 genome evolution, have initiated the continuous sequencing in Bosnia and Herzegovina (Goletic et al., 2021). Persons diagnosed with COVID-19 in Bosnia and Herzegovina are obliged to stay in the self-isolation for 10–14 days or, in case of a severe form of the disease, they are hospitalized. Their household contacts are regarded as “close contacts” and are also quarantined in the households with the recommended precautionary measures. Unlike the usual procedures in Hong Kong where affected pet owners are given the option of having their pets isolated in a designated centre (Sit et al., 2020), pets from affected owners in Bosnia and Herzegovina remain quarantined with them. There is an ongoing project aimed at investigation of SARS-CoV-2 in the animal population which may assist the state public health authorities in determining the best method for managing animals from affected owners. Thirteen dogs and five cats from households with confirmed COVID-19 cases had been tested in the period 01 January—02 March 2021. During this period, three dogs and one cat were found to be infected with the SARS-CoV-2 virus. All tests of human and animal samples, as well as the subsequent genome sequencing, were performed at the Laboratory for molecular-genetic and forensic investigations (LMGFI) of the Veterinary Faculty of the University of Sarajevo, which is the part of BiH public health response to COVID-19 pandemic. This provided us with unique opportunity to test both human and animal samples.

## Materials and Methods

### Data Reporting

No statistical methods were used to determine the sample size. The sampling strategy was organized as a convenient sampling of dogs and cats based on provided verbal or written consent of owners who were previously diagnosed with COVID-19. Given the observational design of the study, the investigators were not blinded to allocation during study and outcome assessment.

### Sample Collection

Samples from dogs and cats were collected by veterinarians from animals sent to the Clinical Center of the Veterinary Faculty of the University of Sarajevo and included nasal, deep oropharyngeal and rectal swabs for RT-qPCR, and blood samples for ELISA and fluorescent immunoassay testing. Samples intended for RT-qPCR were collected using a sterile, soft rayon swab on a plastic shaft with a round bottom plastic pre-labelled tube containing transport medium and kept on cool-packs until arrival in the laboratory. Animals that had a positive test were resampled and retested until no positive PCR results were obtained. Blood sampling was also performed for all thirteen dogs and five cats that participated in the study. The same number of control samples, including nasal, oropharyngeal or rectal swabs, as well as blood samples, were taken from thirteen dogs and five cats that were admitted to Clinical Center of the Veterinary Faculty of the University of Sarajevo and that had no history of COVID-19 nor were ‘close contacts’. Samples from humans were tested using RT-qPCR as part of routine public health response testing for COVID-19 which was performed at the LMGFI of the Veterinary Faculty of the University of Sarajevo. The genetic sequencing, also a part of public health response, was performed at the same institution.

### Serology Assays

All blood samples originating form dogs and cats were tested using ID Screen^®^ SARS-CoV-2 Double Antigen Multi-species ELISA (IDVet, Grabels, France), which screens for antibodies against nucleocapsid SARS-CoV-2 antigen. The samples were prepared and processed as described elsewhere (Sailleau et al., 2020). Moreover, in attempt to distinguish between prior and recent infection, same samples were also tested with AFIAS fluorescent immunoassay (Immunostics Inc., Bioditech, United States), according to the manufacturer’s instructions without modification.

### Quantitative RT-PCR

At the LMGFI, RNA from 140 μL sample from collected swabs was extracted using the QIAamp viral RNA Minikit (Qiagen, Hilden, Germany) or Nextractor^®^ NX-48 Viral RNA kit (Genolution, South Korea) following the manufacturer`s instructions. The extracted samples were eluted into 50 μL. The RT-qPCR for the detection of SARS-CoV-2 was performed on a QuantStudio 5 Real Time PCR System (Applied Biosystems, United States), using a commercial RT-qPCR assay which targets the E, N and ORF1ab gene sequences (XABT Multiple Real-Time PCR Kit for Detection of 2019-nCoV), as well as the protocol described by Corman et al. (2020).

### Sequencing the Viral Genomes

Whole genome sequencing (WGS) of the SARS-CoV-2 genome was done using the ARTIC Network Amplicon sequencing protocol for SARS-CoV-2 v3 without changes to the protocol (Quick, 2020; Tyson et al., 2020). All samples were barcoded and pooled on an R9.4.1. flow cell on a MinION sequencer. The run was terminated after 9 h due to the high genome coverage. Basecalling was done in real-time using the MinIT device, and only the fastq files with the Q score Q > 7 were used for downstream analysis. The bioinformatics analysis was done using the nCoV-2019 novel coronavirus bioinformatics protocol (Loman et al., 2020).

### Phylogenetic Analysis

A dataset of 32 sequences, consisting of the 27 human sequences with the highest sequence identity as the B&H dog/owner sequences, as well as all available whole genome sequences of SARS-CoV-2 virus from dogs at the time (five sequences), was downloaded from GISAID and, along with five B&H dog/owner sequences, used for phylogenetic analysis. All sequences used for generating the phylogenetic tree are listed in Supplementary Table S1. Phylogenetic tree construction was done in MEGAX software using the Maximum Likelihood method (Hasegawa-Kishino-Yano substitution model, 1,000 bootstrap replicates) (Kumar et al., 2018).

## Results

All samples collected from thirteen dogs and five cats with SARS-CoV-2-positive owners, as well as those collected from animals with no recorded “close contacts” were tested and analyzed with both serological assays and RT-qPCR. Only three dogs and one cat yielded positive results, and as such they will be described in detail.

**FIGURE 1:**
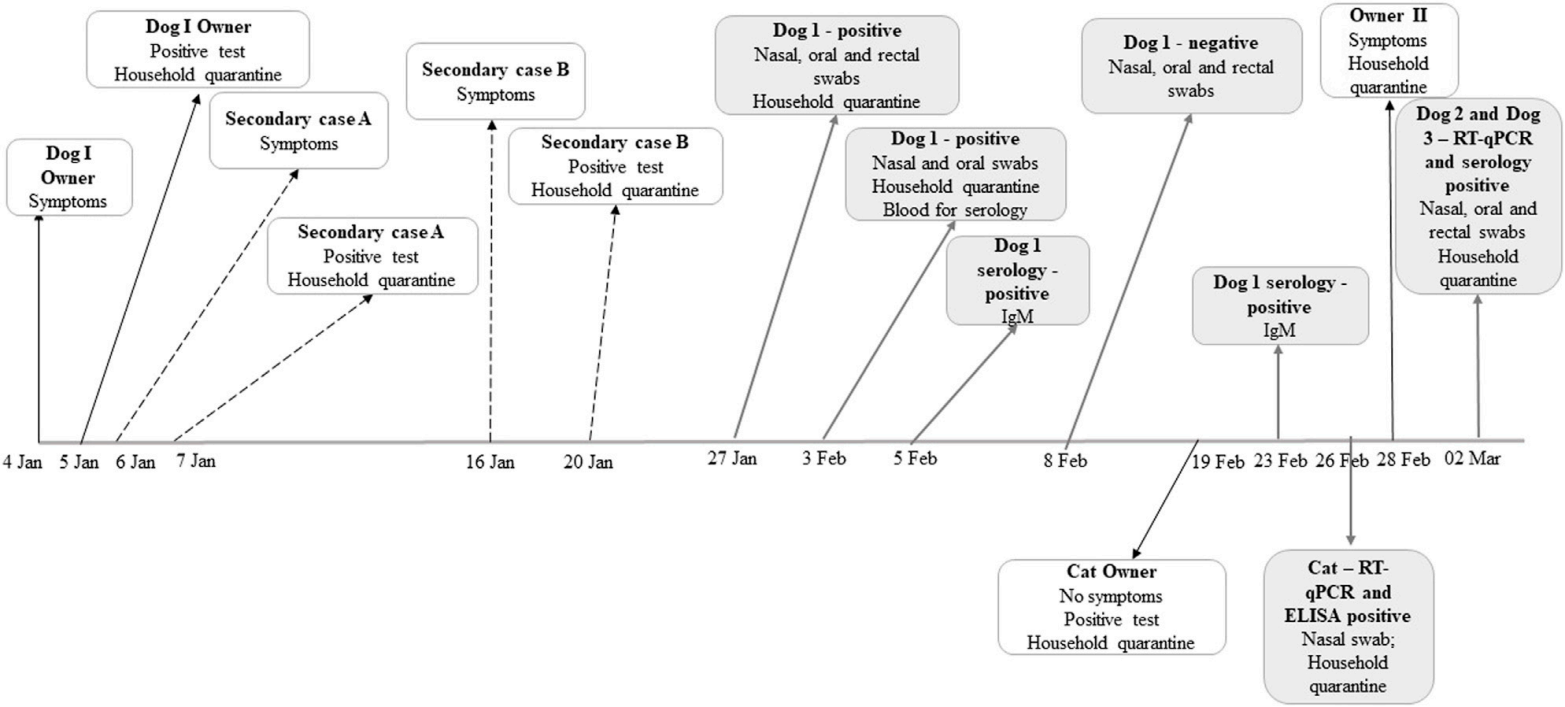
A timeline of events in the human, dog and cat SARS-CoV-2 infection cases that were analyzed in this study.

The infected dog (dog 1) was a 9-year-old neutered female American Staffordshire terrier with previous history of atopic dermatitis, diagnosed at the Clinical Centre of the Veterinary Faculty of the University of Sarajevo. The dog`s owner, a 30-year-old man, developed symptoms on 04 January 2021 and was confirmed to be infected one day later. All members of the household, including the dog, were quarantined, and all tested positive for SARS-CoV-2 for the duration of quarantine (Figure 1 - secondary cases A and B). The nasal, oral and rectal swabs were first collected from the dog on 27 January 2021, after the owner tested negative for SARS-CoV-2. During the quarantine period and clinical examination, the dog showed no obvious clinical signs of the dis-ease. The viral RNA was detected by RT-qPCR in oral and nasal swabs, while no viral RNA could be detected in the rectal swab (Table 1). A subsequent testing (03 February) confirmed these results. Finally, 12 days after initial confirmation, third retesting found no SARS-CoV-2-positive swabs. In addition, serum samples collected consecutively on 05 February and 23 February 2021 were tested seropositive by ELISA. Testing with AFIAS fluorescent immunoassay, on both occasions, produced positive results for IgM antibodies and negative for IgG antibodies. The viral RNA was sequenced directly from the clinical specimens collected from the dog on 27 January and compared with the viral RNA found in clinical specimens from the owner and secondary case B. The full viral genome sequence (29.8 kb) was obtained both from the owner and the dog (GISAID Accession ID, respectively: EPI_ISL_955,184, EPI_ISL_6949571). The viral sequence from the dog was identical to the viral sequence that originated from the own-er across the full genome, which was assigned as a B.1.258 PANGO lineage by GISAID.

**TABLE 1:** RT-qPCR testing results of nasal, oral and rectal swabs of the dogs and cat.

Date of Collection		*Ct* (E)			*Ct* (N)			*Ct* (ORF1ab)		
		Nasal	Oral	Rectal	Nasal	Oral	Rectal	Nasal	Oral	Rectal
Dog 1	27-Jan	25.74	28.26	Neg.	26.01	27.93	Neg.	25.84	27.03	Neg.
	03-Feb	32.45	36.01	Neg.	33.71	37.2	Neg.	32.02	35.98	Neg.
	08-Feb	Neg	Neg.	Neg.	Neg.	Neg.	Neg.	Neg.	Neg.	Neg.
Cat	26-Feb	35.71	Neg.	Neg.	34.98	Neg.	Neg.	34.26	Neg.	Neg.
	03-Mar	Neg.	Neg.	Neg.	Neg.	Neg.	Neg.	Neg.	Neg.	Neg.
Dog 2	02-Mar	14.13	15.03	23.86	12.52	13.98	22.93	15.9	15.55	24.37
	09-Mar	23.49	24	33.81	21.19	23.41	34.23	22.76	24.57	36.11
	15-Mar	35.98	34.77	Neg.	34.63	35.99	Neg.	35.76	33.99	Neg.
	18-Mar	Neg.	Neg.	Neg.	Neg.	Neg.	Neg.	Neg.	Neg.	Neg.
Dog 3	02-Mar	22.57	24.08	36.77	21.36	24.51	Neg.	24.15	23.28	Neg.
	09-Mar	30.14	29.60	Neg	28.97	30.29	Neg.	32.57	31.8	Neg.
	16-Mar	Neg.	Neg.	Neg.	Neg.	Neg.	Neg.	Neg.	Neg.	Neg.
Cut-off Ct for positive		<38	<38	<38	<38	<38	<38	<38	<38	<38

The owner of the second dog (Dog 2, a 4-year-old male Havanese) developed the symptoms on 28 February 2021 and was diagnosed with COVID-19 on 02 March 2021. At that time, the dog was admitted to the Veterinary Faculty showing gastrointestinal problems. Nasal, oral and rectal swabs were first collected from the dog at the admission (02 March 2021), and all tested positive for SARS-CoV-2 viral RNA by RT-qPCR (Table 1). The samples were collected on three more occasions over the span of 17 days, until all swabs tested negative for SARS-CoV-2. Sera samples were seropositive by ELISA, and AFAIS fluorescent immunoassay revealed presence of SARS-CoV-2 IgM antibodies and no IgG antibodies in serum collected at admission. The viral RNA was sequenced directly from the clinical specimens collected from the dog on 02 March (GISAID Accession ID: EPI_ISL_6949572) and compared with the viral RNA found in clinical specimens from the owner (GISAID Accession ID: EPI_ISL_6949577) who re-tested positive on the same day. As in the first confirmed case, the obtained sequences from nasal and rectal swabs were found to be identical across the full genome.

The third dog (Dog 3) was a 6-year-old German Shepherd that was admitted to the Veterinary Faculty and sampled on the same day as Dog 2 (02 March 2021). It was living with the owner that had tested positive for SARS-CoV-2 elsewhere and had been quarantined at the time, so no swabs were taken from the owner. Symptoms noted at the time of admission were sneezing and coughing, and a slight reduction in activity, however these required no veterinary care and the dog recovered within a few days. The samples collected at the day of admission and seven days later tested positive for SARS-CoV-2 by RT-qPCR, but the third RT-qPCR retesting found no evidence of viral RNA (Table 1). Serum samples were detected as seropositive by ELISA, and fluorescent immunoassay AFIAS found IgM antibodies but no IgG antibodies, as is the case with Dog 1 and 2. Sequencing was performed on the samples from 02 March 2021, and the viral sequence was assigned as B.1.1.7 lineage by GISAID (Accession ID: EPI_ISL_5194358).

The infected cat was a 4-year-old Russian blue cat originating from the 26-year-old owner who tested positive for SARS-CoV-2 on 19 February 2021 (see Figure 1) and was asymptomatic. Since the cat was quarantined in the same room as the owner during the obligatory self-isolation, and given the fact that the cat showed mild respiratory signs i.e., coughing and sneezing, it was transferred to the Clinical Center of the Veterinary Faculty on 26 February 2021 where nasal, oral and rectal swabs were collected. SARS-CoV-2 RNA was detected in nasal swab with high Ct values. Specimens that were collected on second occasion (03 March 2021) were negative for the virus. Serum samples were seropositive for ELISA, but negative when tested with AFIAS. All control samples tested negative, either by ELISA or by RT-qPCR.

**FIGURE 2:**
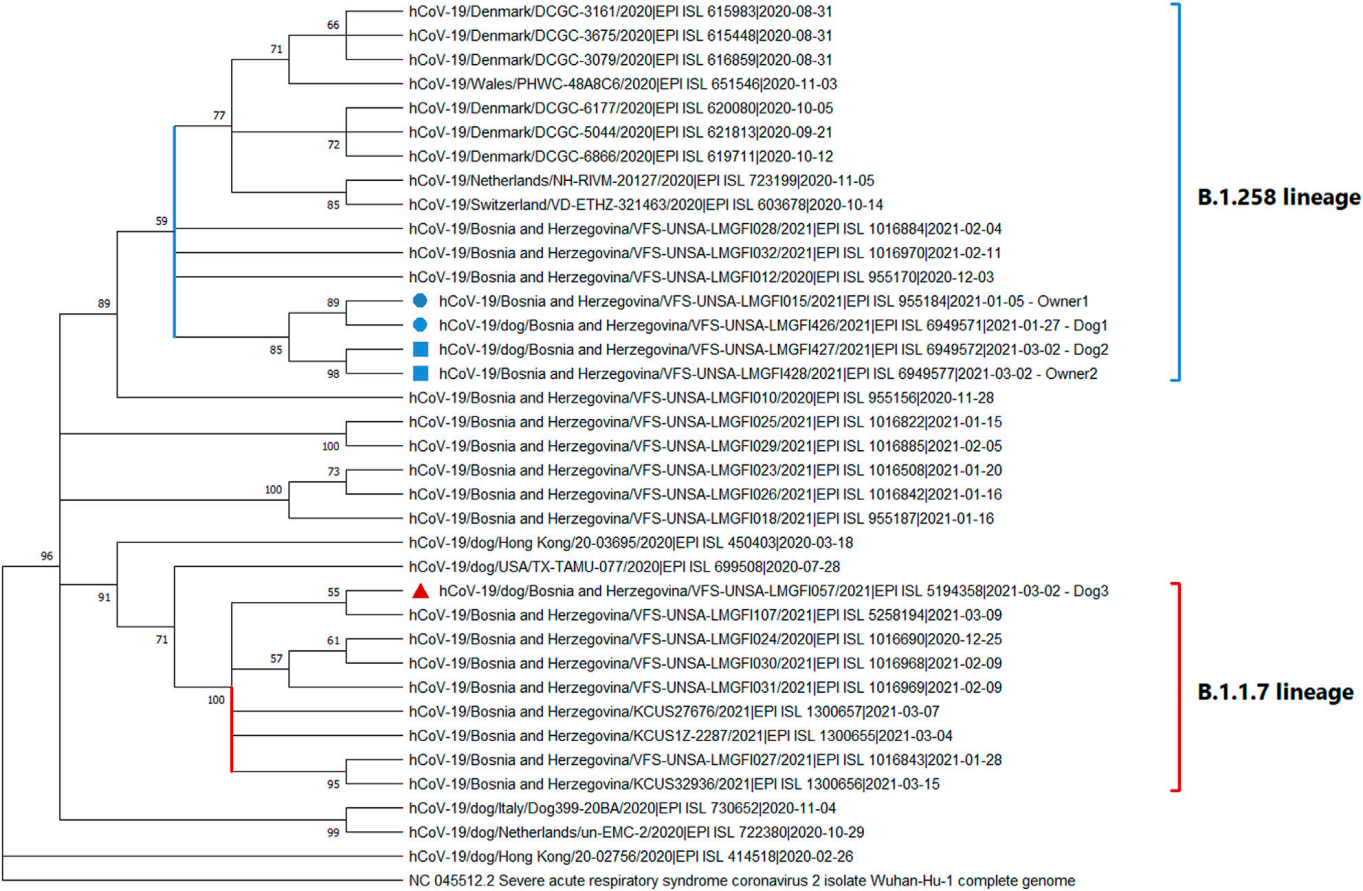
A phylogenetic tree of SARS-CoV-2 showing the clustering of dog and owner virus sequences from Bosnia and Herzegovina. The positions of sequences in the phylogenetic tree are shown with a blue circle for Dog1 and Owner 1 and a blue rectangle for Dog2 and Owner 2 together with other B.1.258 sequences (blue bracket). The sequence of Dog3 is marked with a red triangle, and is found together with other B.1.1.7 sequences (red bracket).

Phylogenetic analysis revealed that the dog/owner sequences 1 and 2 (marked with blue circles and blue rectangles) cluster together with other B&H B.1.258 sequences and form a large group with B.1.258 sequences from other countries (Figure 2, indicated by a blue bracket). The sequences from the two dog/owner cases are clearly distinguishable. Furthermore, sequence from dog 3 (marked with a red triangle) clearly clusters with other B.1.1.7 B&H sequences from the same time period (Figure 2, indicated by a red bracket).

## Discussion

Our results further corroborate the possibility of human-to-animal transmission of SARS-CoV-2 in domestic pets (dogs and a cat) in close contacts with their owners during the obligatory household quarantine. This mode of transmission appears to be very likely as it has recently been demonstrated in a similar study from Hong Kong (Sit et al., 2020). Although there is no clear information on whether the virus can cause illness in pets, our results show a dog with gastrointestinal problems, a dog with slight respiratory problems and a cat with mild symptoms of the infection of the upper respiratory tract. These findings are following the recent study on unusual clinical manifestations in B.1.1.7 infected cats and dogs, including severe cardiac abnormalities secondary to myocarditis and profound impairment of the general health status of the dogs but without any primary respiratory signs (Ferasin et al., 2021). In case of dog 3 however, whose sequence was determined to be a B.1.1.7 lineage, no such severe health impairments were detected and the dog recovered quickly.

Not much is known about the duration of SARS-CoV-2 infection in pets, especially because clinical signs vary or are completely absent. Furthermore, pets are usually not tested in the first place. Thus, the onset of the infection is difficult to ascertain, as is the case with dog 1, whose exact time of infection remains unknown. However, considering the fact that the dog remained quarantined together with positive owner and secondary case A (04 January), the timeline of dog’s infection probably corresponds to the infection of the owner. In line with previous data (Sit et al., 2020) and initial confirmation of dog 1’s positive status on 27 January, observation of relatively low Ct values (Table 1) might suggest prolonged infection or virus shedding. Similar findings were observed in dogs 2 and 3, where detected low Ct values indicate a high viral load. Furthermore, detection of the viral RNA in dog 2 on three occasions over the course of 17 days might shed some light on the duration of SARS-CoV-2 infection in pets. Positive results for IgM antibodies on 05 February 2021 are most likely indication of an acute infection in dog 1. The detection of IgM antibodies and the absence of detectable IgG almost 20 days later (23 February 2021) is an uncommon finding. While indicative, we are aware that results of AFAIS should be treated with care, as it is, to our best knowledge, not validated for use with animal samples.

The finding of B.1.1.7 and B.1.258 infected dogs highlights the possibility that pets may potentially play a relevant role in future SARS-CoV-2 virus dynamic and evolution. To our best knowledge, this is the first recorded case of animal infection with B.1.258 lineage of SARS-CoV-2. Some mutations in B.1.258 lineage, such as N439K and D614G, are reported to be related to host change (Zhang et al., 2020; Thomson et al., 2021), and others, such as P681H, increase transmissibility of the virus (Rambaut et al., 2020); these mutations may have facilitated the transmission of SARS-CoV-2 from human to dog. Interestingly, the D614G and P681H mutations are also found in B.1.1.7, which has also been recorded in cats and dogs (Ferasin et al., 2021). This may also be supporting evidence of the potential role of these mutations in enabling host change and easier transmission of the virus.

## Conclusion

SARS-CoV-2 has been detected in pets and whole genome sequencing has confirmed that the virus originated from the owners that the animals have been quarantined with, and thus in these cases it could be described as an inverse zoonosis. Unusually low Ct values, and developed clinical signs of the disease, may imply pets as more important host to the virus than initially thought. As the ability of the virus to cause the illness in pets and be transmitted back to humans remains an open question, our results warrant the need for further studies aimed at investigating the likelihood of pet-to-human transmission with special emphasis on variants of concern. The low initial Ct values detected underline the need for precautionary measures to protect both humans and animals.

## Data Availability

The datasets presented in this study can be found in online repositories. The names of the repository/repositories and accession number(s) can be found in the article/Supplementary Material.

## References

[B1] Corman V. M. Landt O. Kaiser M. Molenkamp R. Meijer A. Chu D. K. (2020). Detection of 2019 Novel Coronavirus (2019-nCoV) by Real-Time RT-PCR. Euro Surveill. 25, 2000045. 10.2807/1560-7917.es.2020.25.3.2000045 PMC6988269 31992387

[B2] Ferasin L. Fritz M. Ferasin H. Becquart P. Legros V. Leroy E. M. (2021). Myocarditis in Naturally Infected Pets with the British Variant of COVID-19. Vet. Rec. 189, e944. 10.1002/vetr.944 34738231 PMC8661638

[B3] Goletic T. Konjhodzic R. Fejzic N. Goletic S. Eterovic T. Softic A. (2021). Phylogenetic Pattern of SARS-CoV-2 from COVID-19 Patients from Bosnia and Herzegovina: Lessons Learned to Optimize Future Molecular and Epidemiological Approaches. Bosn. J. Basic Med. Sci. 21, 484–487. 10.17305/bjbms.2020.5381 33577445 PMC8292857

[B4] Kiros M. Andualem H. Kiros T. Hailemichael W. Getu S. Geteneh A. (2020). COVID-19 Pandemic: Current Knowledge about the Role of Pets and Other Animals in Disease Transmission. Virol. J. 17, 143. 10.1186/s12985-020-01416-9 33008410 PMC7530550

[B5] Kumar S. Stecher G. Li M. Knyaz C. Tamura K. (2018). MEGA X: Molecular Evolutionary Genetics Analysis across Computing Platforms. MEGA X: Mol. Evol. Genet. Anal. across Comput. Platforms. Mol. Biol. Evol. 35, 1547–1549. 10.1093/molbev/msy096 PMC5967553 29722887

[B6] Loman N. Rowe W. Rambaut A. (2020). nCoV-2019 Novel Coronavirus Bioinformatics Protocol. Available at: https://artic.network/ncov-2019/ncov2019-bioinformatics-sop.html (Accessed May 03, 2021).

[B7] Quick J. (2020). nCoV-2019 Sequencing Protocol V3 (LoCost). Available at: https://protocols.io/view/ncov-2019-sequencing-protocol-v3-locost-bh42j8ye (Accessed May 03, 2021).

[B8] Rambaut A. Loman N. Pybus O. Barclay W. Barrett J. Carabelli A. (2020). (CoG-UK) Preliminary Genomic Characterization of an Emergent SARS-CoV-2 Lineage in the UK Defined by a Novel Set of Spike Mutations. Virological.org. Available at: https://virological.org/t/preliminary-genomic-characterisation-of-an-emergent-sars-cov-2-lineage-in-the-uk-defined-by-a-novel-set-of-spike-mutations/563 (Accessed Apr 30, 2021).

[B9] Sailleau C. Dumarest M. Vanhomwegen J. Delaplace M. Caro V. Kwasiborski A. (2020). First Detection and Genome Sequencing of SARS‐CoV‐2 in an Infected Cat in France. Transbound. Emerg. Dis. 67, 2324–2328. 10.1111/tbed.13659 32500944 PMC7300955

[B10] Sit T. H. C. Brackman C. J. Ip S. M. Tam K. W. S. Law P. Y. T. To E. M. W. (2020). Infection of Dogs with SARS-CoV-2. Nature 586, 776–778. 10.1038/s41586-020-2334-5 32408337 PMC7606701

[B11] Thomson E. C. Rosen L. E. Shepherd J. G. Spreafico R. da Silva Filipe A. Wojcechowskyj J. A. (2021). Circulating SARS-CoV-2 Spike N439K Variants Maintain Fitness while Evading Antibody-Mediated Immunity. Cell. 184, 1171–1187. 10.1016/j.cell.2021.01.037 33621484 PMC7843029

[B12] Tyson J. R. James P. Stoddart D. Sparks N. Wickenhagen A. Hall G. (2020). Improvements to the ARTIC Multiplex PCR Method for SARS-CoV-2 Genome Sequencing Using Nanopore (Preprint). bioRxiv, 1–19. 10.1101/2020.09.04.283077

[B13] World Health Organisation (2021). Weekly Epidemiological Update on COVID-19. [cited 2021 December 03]. Available at: https://www.who.int/publications/m/item/weekly-epidemiological-update-on-covid-19–-30-november-2021 .

[B14] Zhang L. Jackson C. B. Mou H. Ojha A. Peng H. Quinlan B. D. (2020). SARS-CoV-2 Spike-Protein D614G Mutation Increases Virion Spike Density and Infectivity. Nat. Commun. 11, 6013. 10.1038/s41467-020-19808-4 33243994 PMC7693302

[B15] Zhu N. Zhang D. Wang W. Li X. Yang B. Song J. (2020). A Novel Coronavirus from Patients with Pneumonia in China, 2019. N. Engl. J. Med. 382, 727–733. 10.1056/NEJMoa2001017 31978945 PMC7092803

