# Supplementary material for: The Evidence of SARS-CoV-2 Human-to-Pets Transmission in Household Settings in Bosnia and Herzegovina
Source: Front Genet. 2022 Apr 26;13:839205. doi: 10.3389/fgene.2022.839205 (PMC9086552; doi:10.3389/fgene.2022.839205)
Supplement: Supplementary file 1 [file Table1.DOCX]

Supplementary Material

**Supplementary Table 1.** Background information of sequences used to generate the phylogenetic tree.

| **Virus name** | **Accession ID** | **Collection date** | **Host** | **PANGO lineage** | **Location** | **Originating laboratory** | **Submitting laboratory** | **Authors** |
| --- | --- | --- | --- | --- | --- | --- | --- | --- |
| hCoV-19/Switzerland/VD-ETHZ-321463/2020 | EPI_ISL_603678 | 14/10/2020 | Human | B.1.258 | Switzerland | Viollier AG | Department of Biosystems Science and Engineering, ETH Zürich | Beisel C. et al. |
| hCoV-19/Denmark/DCGC-3675/2020 | EPI_ISL_615448 | 31/08/2020 | Human | B.1.258 | Denmark | Department of Virus and Microbiological Special Diagnostics, Statens Serum Institut, Denmark | Albertsen lab, Department of Chemistry and Bioscience, Aalborg University, Denmark | Danish Covid-19 Genome Consortia |
| hCoV-19/Denmark/DCGC-3161/2020 | EPI_ISL_615983 | 31/08/2020 | Human | B.1.258 | Denmark | Department of Virus and Microbiological Special Diagnostics, Statens Serum Institut, Denmark | Albertsen lab, Department of Chemistry and Bioscience, Aalborg University, Denmark | Danish Covid-19 Genome Consortia |
| hCoV-19/Denmark/DCGC-3079/2020 | EPI_ISL_616859 | 31/08/2020 | Human | B.1.258 | Denmark | Department of Virus and Microbiological Special Diagnostics, Statens Serum Institut, Denmark | Albertsen lab, Department of Chemistry and Bioscience, Aalborg University, Denmark | Danish Covid-19 Genome Consortia |
| hCoV-19/Denmark/DCGC-6866/2020 | EPI_ISL_619711 | 12/10/2020 | Human | B.1.258 | Denmark | Department of Virus and Microbiological Special Diagnostics, Statens Serum Institut, Denmark | Albertsen lab, Department of Chemistry and Bioscience, Aalborg University, Denmark | Danish Covid-19 Genome Consortia |
| hCoV-19/Denmark/DCGC-6177/2020 | EPI_ISL_620080 | 05/10/2020 | Human | B.1.258 | Denmark | Department of Virus and Microbiological Special Diagnostics, Statens Serum Institut, Denmark | Albertsen lab, Department of Chemistry and Bioscience, Aalborg University, Denmark | Danish Covid-19 Genome Consortia |
| hCoV-19/Denmark/DCGC-5044/2020 | EPI_ISL_621813 | 21/09/2020 | Human | B.1.258 | Denmark | Department of Virus and Microbiological Special Diagnostics, Statens Serum Institut, Denmark | Albertsen lab, Department of Chemistry and Bioscience, Aalborg University, Denmark | Danish Covid-19 Genome Consortia |
| hCoV-19/Wales/PHWC-48A8C6/2020 | EPI_ISL_651546 | 03/11/2020 | Human | B.1.258.7 | United Kingdom / Wales | Wales Specialist Virology Centre Sequencing lab: Pathogen Genomics Unit | COVID-19 Genomics UK (COG-UK) Consortium | Moore C. et al. |
| hCoV-19/Netherlands/NH-RIVM-20127/2020 | EPI_ISL_723199 | 05/11/2020 | Human | B.1.258 | Netherlands | Dutch COVID-19 response team | National Institute for Public Health and the Environment (RIVM) | Meijer A. et al. |
| hCoV-19/Bosnia and Herzegovina/VFS-UNSA-LMGFI015/2021 | EPI_ISL_955184 | 05/01/2021 | Human | B.1.258 | Bosnia and Herzegovina | University of Sarajevo, Veterinary Faculty, Laboratory for Molecular Diagnostic and Research Laboratory | University of Sarajevo, Veterinary Faculty, Laboratory for Molecular Diagnostic and Research Laboratory | Goletic S. et al. |
| hCoV-19/Bosnia and Herzegovina/VFS-UNSA-LMGFI028/2021 | EPI_ISL_1016884 | 04/02/2021 | Human | B.1.258.17 | Bosnia and Herzegovina | University of Sarajevo, Veterinary Faculty, Laboratory for Molecular Diagnostic and Research Laboratory | University of Sarajevo, Veterinary Faculty, Laboratory for Molecular Diagnostic and Research Laboratory | Goletic T. et al. |
| hCoV-19/Bosnia and Herzegovina/VFS-UNSA-LMGFI032/2021 | EPI_ISL_1016970 | 11/02/2021 | Human | B.1.258 | Bosnia and Herzegovina | University of Sarajevo, Veterinary Faculty, Laboratory for Molecular Diagnostic and Research Laboratory | University of Sarajevo, Veterinary Faculty, Laboratory for Molecular Diagnostic and Research Laboratory | Goletic S. et al. |
| hCoV-19/Bosnia and Herzegovina/VFS-UNSA-LMGFI012/2020 | EPI_ISL_955170 | 03/12/2020 | Human | B.1.258 | Bosnia and Herzegovina | University of Sarajevo, Veterinary Faculty, Laboratory for Molecular Diagnostic and Research Laboratory | University of Sarajevo, Veterinary Faculty, Laboratory for Molecular Diagnostic and Research Laboratory | Goletic T. et al. |
| hCoV-19/dog/Bosnia and Herzegovina/VFS-UNSA-LMGFI426/2021 | EPI_ISL_6949571 | 27/01/2021 | *Canis lupus familiaris* | B.1.258 | Bosnia and Herzegovina | University of Sarajevo, Veterinary Faculty, Laboratory for Molecular Diagnostic and Research Laboratory | University of Sarajevo, Veterinary Faculty, Laboratory for Molecular Diagnostic and Research Laboratory | Goletic S. et al. |
| hCoV-19/dog/Bosnia and Herzegovina/VFS-UNSA-LMGFI427/2021 | EPI_ISL_6949572 | 02/03/2021 | *Canis lupus familiaris* | B.1.258 | Bosnia and Herzegovina | University of Sarajevo, Veterinary Faculty, Laboratory for Molecular Diagnostic and Research Laboratory | University of Sarajevo, Veterinary Faculty, Laboratory for Molecular Diagnostic and Research Laboratory | Goletic S. et al. |
| hCoV-19/Bosnia and Herzegovina/VFS-UNSA-LMGFI428/2021 | EPI_ISL_6949577 | 02/03/2021 | Human | B.1.258 | Bosnia and Herzegovina | University of Sarajevo, Veterinary Faculty, Laboratory for Molecular Diagnostic and Research Laboratory | University of Sarajevo, Veterinary Faculty, Laboratory for Molecular Diagnostic and Research Laboratory | Goletic S. et al. |
| hCoV-19/Bosnia and Herzegovina/VFS-UNSA-LMGFI010/2020 | EPI_ISL_955156 | 28/11/2020 | Human | B.1.258 | Bosnia and Herzegovina | University of Sarajevo, Veterinary Faculty, Laboratory for Molecular Diagnostic and Research Laboratory | University of Sarajevo, Veterinary Faculty, Laboratory for Molecular Diagnostic and Research Laboratory | Goletic T. et al. |
| hCoV-19/Bosnia and Herzegovina/VFS-UNSA-LMGFI025/2021 | EPI_ISL_1016822 | 15/01/2021 | Human | B.1.160 | Bosnia and Herzegovina | University of Sarajevo, Veterinary Faculty, Laboratory for Molecular Diagnostic and Research Laboratory | University of Sarajevo, Veterinary Faculty, Laboratory for Molecular Diagnostic and Research Laboratory | Goletic T. et al. |
| hCoV-19/Bosnia and Herzegovina/VFS-UNSA-LMGFI029/2021 | EPI_ISL_1016885 | 05/02/2021 | Human | B.1.160 | Bosnia and Herzegovina | University of Sarajevo, Veterinary Faculty, Laboratory for Molecular Diagnostic and Research Laboratory | University of Sarajevo, Veterinary Faculty, Laboratory for Molecular Diagnostic and Research Laboratory | Goletic S. et al. |
| hCoV-19/Bosnia and Herzegovina/VFS-UNSA-LMGFI023/2021 | EPI_ISL_1016508 | 20/01/2021 | Human | B.1.565 | Bosnia and Herzegovina | University of Sarajevo, Veterinary Faculty, Laboratory for Molecular Diagnostic and Research Laboratory | University of Sarajevo, Veterinary Faculty, Laboratory for Molecular Diagnostic and Research Laboratory | Goletic T. et al. |
| hCoV-19/Bosnia and Herzegovina/VFS-UNSA-LMGFI026/2021 | EPI_ISL_1016842 | 16/01/2021 | Human | B.1.565 | Bosnia and Herzegovina | University of Sarajevo, Veterinary Faculty, Laboratory for Molecular Diagnostic and Research Laboratory | University of Sarajevo, Veterinary Faculty, Laboratory for Molecular Diagnostic and Research Laboratory | Goletic T. et al. |
| hCoV-19/Bosnia and Herzegovina/VFS-UNSA-LMGFI018/2021 | EPI_ISL_955186 | 16/01/2021 | Human | B.1.565 | Bosnia and Herzegovina | University of Sarajevo, Veterinary Faculty, Laboratory for Molecular Diagnostic and Research Laboratory | University of Sarajevo, Veterinary Faculty, Laboratory for Molecular Diagnostic and Research Laboratory | Goletic S. et al. |
| hCoV-19/dog/Bosnia and Herzegovina/VFS-UNSA-LMGFI057/2021 | EPI_ISL_5194358 | 02/03/2021 | *Canis lupus familiaris* | B.1.1.7 | Bosnia and Herzegovina | University of Sarajevo, Veterinary Faculty, Laboratory for Molecular Diagnostic and Research Laboratory | University of Sarajevo, Veterinary Faculty, Laboratory for Molecular Diagnostic and Research Laboratory | Goletic S. et al. |
| hCoV-19/Bosnia and Herzegovina/VFS-UNSA-LMGFI107/2021 | EPI_ISL_5258194 | 09/03/2021 | Human | B.1.1.7 | Bosnia and Herzegovina | University of Sarajevo, Veterinary Faculty, Laboratory for Molecular Diagnostic and Research Laboratory | University of Sarajevo, Veterinary Faculty, Laboratory for Molecular Diagnostic and Research Laboratory | Goletic T. et al. |
| hCoV-19/Bosnia and Herzegovina/KCUS27676/2021 | EPI_ISL_1300657 | 07/03/2021 | Human | B.1.1.7 | Bosnia and Herzegovina | Clinical Center, University of Sarajevo; Unit for Clinical Microbiology | Clinical Center, University of Sarajevo; Unit for Clinical Microbiology | Salimovic-Besic I. et al. |
| hCoV-19/Bosnia and Herzegovina/KCUS1Z-2287/2021 | EPI_ISL_1300655 | 04/03/2021 | Human | B.1.1.7 | Bosnia and Herzegovina | Clinical Center, University of Sarajevo; Unit for Clinical Microbiology | Clinical Center, University of Sarajevo; Unit for Clinical Microbiology | Salimovic-Besic I. et al. |
| hCoV-19/Bosnia and Herzegovina/VFS-UNSA-LMGFI027/2021 | EPI_ISL_1016843 | 28/01/2021 | Human | B.1.1.7 | Bosnia and Herzegovina | University of Sarajevo, Veterinary Faculty, Laboratory for Molecular Diagnostic and Research Laboratory | University of Sarajevo, Veterinary Faculty, Laboratory for Molecular Diagnostic and Research Laboratory | Goletic T. et al. |
| hCoV-19/Bosnia and Herzegovina/KCUS32936/2021 | EPI_ISL_1300656 | 15/03/2021 | Human | B.1.1.7 | Bosnia and Herzegovina | Clinical Center, University of Sarajevo; Unit for Clinical Microbiology | Clinical Center, University of Sarajevo; Unit for Clinical Microbiology | Salimovic-Besic I. et al. |
| hCoV-19/Bosnia and Herzegovina/VFS-UNSA-LMGFI031/2022 | EPI_ISL_1016969 | 09/02/2021 | Human | B.1.1.7 | Bosnia and Herzegovina | University of Sarajevo, Veterinary Faculty, Laboratory for Molecular Diagnostic and Research Laboratory | University of Sarajevo, Veterinary Faculty, Laboratory for Molecular Diagnostic and Research Laboratory | Goletic S. et al. |
| hCoV-19/Bosnia and Herzegovina/VFS-UNSA-LMGFI024/2020 | EPI_ISL_1016690 | 25/12/2020 | Human | B.1.1.7 | Bosnia and Herzegovina | University of Sarajevo, Veterinary Faculty, Laboratory for Molecular Diagnostic and Research Laboratory | University of Sarajevo, Veterinary Faculty, Laboratory for Molecular Diagnostic and Research Laboratory | Goletic T. et al. |
| hCoV-19/Bosnia and Herzegovina/VFS-UNSA-LMGFI030/2021 | EPI_ISL_1016968 | 09/02/2021 | Human | B.1.1.7 | Bosnia and Herzegovina | University of Sarajevo, Veterinary Faculty, Laboratory for Molecular Diagnostic and Research Laboratory | University of Sarajevo, Veterinary Faculty, Laboratory for Molecular Diagnostic and Research Laboratory | Goletic S. et al. |
| hCoV-19/dog/Hong Kong/20-03695/2020 | EPI_ISL_450403 | 18/03/2020 | *Canis lupus familiaris* | B.1.1 | Hong Kong | School of Public Health, The University of Hong Kong | School of Public Health, The University of Hong Kong | Sit T.H.S. et al. |
| hCoV-19/dog/USA/TX-TAMU-077/2020 | EPI_ISL_699508 | 28/07/2020 | *Canis lupus familiaris* | B.1.1 | USA | Diagnostic Virology Laboratory, USDA National Veterinary Services Laboratories | Diagnostic Virology Laboratory, USDA National Veterinary Services Laboratories | Hamer S.A. et al. |
| hCoV-19/dog/Italy/Dog399-20BA/2020 | EPI_ISL_730652 | 04/11/2020 | *Canis lupus familiaris* | B.1.177 | Italy | University of Bari, Valenzano, Italy | Dept. Food safety, Nutrition and Veterinary Public Health, Istituto superiore di sanità | Decaro N. et al. |
| hCoV-19/dog/Netherlands/un-EMC-2/2020 | EPI_ISL_722380 | 29/10/2020 | *Canis lupus familiaris* | B.1.177 | Netherlands | Dutch COVID-19 response team | Erasmus Medical Center | Munnink B.O. et al. |
| hCoV-19/dog/Hong Kong/20-02756/2020 | EPI_ISL_414518 | 26/02/2020 | *Canis lupus familiaris* | B.43 | Hong Kong | Tai Lung Veterinary Laboratory, Agriculture, Fisheries and Conservation Department | School of Public Health, The University of Hong Kong | Chung T.S.H. et al. |
| Severe acute respiratory syndrome coronavirus 2 isolate Wuhan-Hu-1, complete genome | NC_045512.2 (NCBI Accession number) | 05/01/2020 (first submitted) | Human | / | China | Shanghai Public Health Clinical Center & School of Public Health, Fudan University, Shanghai, China | Shanghai Public Health Clinical Center & School of Public Health, Fudan University, Shanghai, China | Wu F. et al. |
